# Identification and solution of drug-related problems in the neurology unit of a tertiary hospital in China

**DOI:** 10.1186/s40360-021-00530-w

**Published:** 2021-10-26

**Authors:** Pengpeng Liu, Guangyao Li, Mei Han, Chao Zhang

**Affiliations:** 1 Department of Pharmacy, Beijing Tongren Hospital, Capital Medical University, 1 Dongjiaominxiang Street, Dongcheng District, Beijing, 100730 China; 2 Evidence-Based Medicine Center, Beijing University of Chinese Medicine, Beijing, China

**Keywords:** Drug-related problems, Neurology unit, PCNE, Pharmacists’ intervention

## Abstract

**Background:**

The prevalence and characteristics of drug-related problems (DRPs) and factors associated with the occurrence of DRPs in the neurology unit in China remain unknown. This study aimed to determine the prevalence, characteristics and severity ratings of DRPs and identify factors associated with the occurrence of DRPs in the neurology unit of a tertiary care and academic teaching hospital in China.

**Methods:**

A retrospective study of DRPs and pharmacists’ interventions for neurology patients was performed during a non-consecutive 24-month study period. Patient demographics and clinical characteristics, and pharmacist’s intervention records were collected. The characteristics and severity ratings of DRPs were categorized using the Pharmaceutical Care Network Europe (PCNE) DRP classification tool V9.00 and the National Coordinating Council for Medication Error Reporting and Prevention (NCC-MERP) classification respectively.

**Results:**

A total of 242 DRPs were detected for 974 admitted patients, an average of 0.25 DRPs per patient. Treatment safety was the major type of DRPs (106;43.8%) followed by treatment effectiveness (78;32.2%). The primary causes of DRPs were drug selection (124;44.1%) and dose selection (92;32.7%). Clinical pharmacists provided 525 interventions, and most interventions occurred at the prescriber level (241;45.9%). A total of 91.4% of these interventions were accepted, contributing to solving 93.0% of the identified problems. The majority of DRPs (210;86.8%) were rated at severity categories B to D (causing no patient harm). Multiple logistic regression showed that creatinine clearance, number of medications used, nasogastric feeding, diabetes, and infectious diseases were associated with more frequent DRPs (*p* < 0.05).

**Conclusions:**

DRPs are relatively common in the neurology unit in China, with primary causes of drug and dose selection, and clinical pharmacists can effectively reduce and prevent DRPs to optimize medication therapy.

## Background

Globally, neurological disorders were the leading cause of disability and the second leading cause of death [1]. Neurological disorders are frequent in the general population, especially in older adults, and most patients are accompanied by other chronic diseases, requiring the combined application of multiple medications, which increases the incidence of drug-related problems (DRPs) [2, 3]. The concept of DRPs is defined as ‘an event or circumstance involving drug therapy that actually or potentially interferes with desired health outcomes’ [4]. DRPs include medication errors (MEs), adverse drug events (ADEs) [5]. Krähenbühl-Melcher found that approximately 8% of hospitalized patients experience an ADE, and 5–10% of medication prescriptions are erroneous in a systematic review of the years from 1990 to 2005 [6]. DRPs occur for many reasons, for example, inappropriate combination of medications, inappropriate medication form, and medication dose too high, leading to an increase in morbidity, mortality, and medical costs [4–6].

The prevention, identification and solution of DRPs constitutes the core of pharmaceutical care in which clinical pharmacists, together with the multi-disciplinary team (MDT), make an effort to improve therapeutic outcomes and the quality of life in patients [7–9]. Clinical pharmacists are involved in the entire treatment process from the beginning of patient hospitalization including medication reconciliation, participating in clinical ward rounds, providing medication consultation and so on. By monitoring DRPs and carrying out the appropriate interventions, clinical pharmacists play an increasingly important role in improving the efficacy and safety of medication therapy [10–14].

Studies from different countries discovered an average of 0.29–1.45 DRPs per patient admitted into the neurology unit [15–18]. These studies further indicate that clinical pharmacists can effectively identify and resolve DRPs in patients with neurological diseases. However, the prevalence and characteristics of DRPs in patients admitted into the neurology unit in China and factors associated with the occurrence of DRPs in this population are largely unknown.

## Methods

### Setting and study design

This retrospective study was performed in the 21-bed neurology unit on patients hospitalized from Apr 1, 2018 to Sep 31, 2020 at the Southern District of Beijing Tongren Hospital affiliated to Capital Medical University, a 1759-bed tertiary care, teaching and researching institution. Patients admitted into the neurology unit were cared for by a MDT, including a clinical pharmacist. The clinical pharmacist had obtained clinical pharmacy training certificates from the China Ministry of Health and had 5 years of hospital practice experience.

### Data collection

Patients who required at least one overnight stay in the neurology unit were recruited during the study period. This period from Jan 1, 2020 to Jun 30, 2020 was not included in the study considering the effect of the novel coronavirus disease 2019 (COVID-19) on hospitalization rates. The clinical activities of the clinical pharmacist were: (1) reviewing medication orders to identify DRPs and proposing clinical interventions to resolve the DRPs identified, (2) visited the patients within 24-72 h of patient admission to the ward and medication reconciliation, (3) participating in daily MDT ward round and providing therapy advice to the MDT, (4) detecting and reporting adverse drug reactions, (5) providing discharge education. All DRPs identified by clinical pharmacists were documented, categorized, and entered into a data collection sheet. The sheet included patient clinical characteristics, types and causes of DRPs, pharmacists’ interventions, outcomes of interventions, and screenshots of DRPs. The following patient demographics and clinical information were collected: gender, age, smoking and drinking habits, body mass index (BMI), length of hospital stay, patient admission diagnosis, concomitant diseases, creatinine clearance, types of medications used, nasogastric feeding, number of medications used.

The identified DRPs, causes, interventions, and outcomes were categorized and characterized using the Pharmaceutical Care Network Europe (PCNE) DRP classification (version 9.00) system, which was last updated in 2019 [4]. The PCNE DRP classification system is a validated DRP classification used in a variety of settings, and it includes five domains: problems (P), causes (C), planned interventions (I), intervention acceptance (A), and status of the DRP (O). Although one problem may have multiple causes and lead to more than one intervention, it leads to only one outcome. The severity ratings of the outcomes of DRPs were categorized using the National Coordinating Council for Medication Error Reporting and Prevention (NCC-MERP) classification [19]. This classification comprises four categories according to ascending severity of the patient outcome: (1) circumstances or events that have the capacity to cause error (no error, subcategory A); (2) MEs occurred without posing harm to patients (subcategories B, C and D); (3) MEs caused harm to patients (subcategories E, F, G and H); (4) MEs resulted in a patient’s death (subcategory I). The severity ratings of DRPs were performed by two pharmacists independently.

### Statistical analysis

We divided the patients into DRP and non-DRP groups. A descriptive analysis was performed on the patient’s demographics, clinical characteristics, identified DRPs, causes of DRPs, and types and outcomes of interventions. The Student’s t-test for continuous variables was used to compare means between groups when continuous variables conformed to the homogeneity of variance and normal distribution. Otherwise, the Mann-Whitney U test was used, represented by medians and interquartile ranges (IQRs) (25-75th percentiles). Categorical variables were represented by frequencies and percentages, and between-group differences were analyzed using the Chi-square test and Fisher’s exact test if necessary. Variance inflation factor (VIF) values were calculated to measure the degree of multicollinearity among the variables that were significant in the univariate analysis (*p* < 0.1). A VIF of > 10 was considered indicative of multicollinearity and excluded from the logistic regression analysis. Based on the univariate analysis and VIF values, variables that were significant (*p* < 0.1) were included in the multiple logistic regression analysis to identify factors associated with the occurrence of DRPs. All statistical analyses were carried out using SPSS (Version 27.0). *p* values < 0.05 were considered statistically significant.

## Results

### Patient characteristics

During the 24-month study period, 1225 patients were admitted to the neurology unit, 251 patients of them were excluded including 154 patients with less than one night, 95 patients admitting from Jan 2020 to Jun 2020, and 2 patients with no medication therapy. A total of 974 patients were eligible, and 198 (20.3%) patients had at least one DRP requiring pharmacist interventions. The median age of the study patients was 62.0(54.0,70.0) years and 65.9% were males. According to the International Classification of Diseases 10 (ICD-10), admitted patients suffered mainly from cerebrovascular disease, disorders of the optic nerve and visual pathways, episodic and paroxysmal disorders. The most common comorbidities were hyperlipidemia (82.9%), hypertension (70.2%), diabetes (35.8%), gastrointestinal diseases (28.6%), hyperhomocysteinemia (15.8%). Tables 1 and 2 provide the baseline characteristics of all patients and DRP status.

**Table 1:** Patient demographic and disease characteristics

Number	Total 974 (100%)	With DRP 198 (20.3%)	Without DRP 776 (79.7%)
Characteristics			
Sex, male	642 (65.9)	134 (67.7)	508 (65.5)
Age	62.0 (54.0,70.0)	64.0 (55.0,73.0)	62.0 (53.0,69.0) **
Smoke, currently	353 (36.2)	82 (41.4)	271 (34.9)
Alcohol, currently	297 (30.5)	58 (29.3)	239 (30.8)
Body mass index (kg/m2)	25.2 (23.1,27.5)	25.4 (23.5,26.9)	25.2 (23.0,27.6)
Length of hospital stay, days	14.0 (11.0,15.0)	14.0 (11.0,15.0)	13.0 (11.0,15.0)
Admission diagnosis			
Cerebrovascular diseases ^a^	704 (72.3)	156 (78.8)	548 (70.6) *
Episodic and paroxysmal disorders ^b^	64 (6.6)	11 (5.6)	53 (6.8)
Disorders of the optic nerve and visual pathways ^c^	65 (6.7)	8 (4.0)	57 (7.3)
Demyelinating diseases of the central nervous system ^d^	21 (2.2)	4 (2.0)	17 (2.2)
Paralytic strabismus ^e^	22 (2.3)	3 (1.5)	19 (2.4)
Nerve, nerve root, and plexus disorders ^f^	10 (1.0)	3 (1.5)	7 (0.9)
Parkinson disease	5 (0.5)	2 (1.0)	3 (0.4)
Myasthenia gravis	21 (2.2)	1 (0.5)	20 (2.6)
Inflammatory diseases of the central nervous system ^g^	10 (1.0)	2 (1.0)	8 (1.0)
Polyneuropathies and other disorders of the peripheral nervous system ^h^	7 (0.7)	1 (0.5)	6 (0.8)
Others	32 (3.3)	7 (3.5)	25 (3.2)
Concomitant diseases			
Hyperlipidemia	807 (82.9)	168 (84.8)	639 (82.3)
Hypertension	684 (70.2)	156 (78.8)	528 (68.0) **
Diabetes	349 (35.8)	99 (50.0)	250 (32.2) ***
Digestive system diseases	279 (28.6)	67 (33.8)	212 (27.3)
Hyperhomocysteinemia (HCY)	154 (15.8)	38 (19.2)	116 (14.9)
Liver dysfunction	128 (13.1)	34 (17.2)	94 (12.1)
Coronary heart disease	127 (13.0)	25 (12.6)	102 (13.1)
Sleep disorder	107 (11.0)	26 (13.1)	81 (10.4)
Creatinine clearance (ml/min)	101.7 (80.3127.9)	94.6 (74.9121.4)	103.3 (80.8129.1) *
Respiratory diseases	87 (8.9)	19 (9.6)	68 (8.8)
Infectious diseases	74 (7.6)	36 (18.2)	38 (4.9) ***
Hyperuricemia/gout	70 (7.2)	19 (9.6)	51 (6.6)
Benign prostatic hyperplasia (BPH)	70 (7.2)	17 (8.6)	53 (6.8)
Peripheral neuropathy	68 (7.0)	13 (6.6)	55 (7.1)
Anxiety-depressive state	41 (4.2)	10 (5.1)	31 (4.0)
Deep venous thrombosis (DVT)	37 (3.8)	12 (6.1)	25 (3.2)
Atrial fibrillation	26 (2.7)	13 (6.6)	13 (1.7) ***

*p < 0.05; ** *p* < 0.005; *** *p* < 0.0001

^a^Cerebral infarction, cerebral ischemia, cerebral haemorrhage; ^b^ Transient ischemic attack, epilepsy, migraine; ^c^ Anterior ischemic optic neuropathy, optic neuritis; ^d^ Multiple sclerosis, neuromyelitis optica spectrum disease; ^e^ Abducens nerve paralysis; ^f^ Diabetic mononeuropathy, trigeminal paralysis; ^g^ Purulent meningoencephalitis, nonspecific cavernous sinusitis; ^h^ Ischemic peripheral neuropathy, Guillain-Barre syndrome

**Table 2:** Baseline medication class in patients

Number	Total 974 (100%)	With DRP 198 (20.3%)	Without DRP 776 (79.7%)
Medication			
Antiplatelet agents	798 (81.9)	171 (86.4)	627 (80.8)
Anticoagulants	44 (4.5)	18 (9.1)	26 (3.4) **
Antihyperlipidemic agents	842 (86.4)	177 (89.4)	665 (85.7)
Antihypertensive agents	692 (71.0)	165 (83.3)	527 (67.9) ***
Antidiabetic agents	318 (32.6)	90 (45.5)	228 (29.4) ***
Glucocorticoids	96 (9.9)	17 (8.6)	79 (10.2)
Antiepileptic medications	10 (1.0)	2 (1.0)	8 (1.0)
Vitamins	258 (26.5)	47 (23.7)	211 (27.2)
Treatment of prostatic hyperplasia	35 (3.6)	8 (4.0)	27 (3.5)
Digestive system medications	450 (46.2)	103 (52.0)	347 (44.7)
Electrolytes	192 (19.7)	49 (24.7)	143 (18.4) *
TCM medications	830 (85.2)	173 (87.4)	657 (84.7)
Respiratory medications	93 (9.5)	29 (14.6)	64 (8.2) *
Antianxiety or Antidepressant	39 (4.0)	10 (5.1)	29 (3.7)
Sedative hypnotics	108 (11.1)	26 (13.1)	82 (10.6)
Anti-infective medications	93 (9.5)	37 (18.7)	56 (7.2) ***
Liver protective medications	113 (11.6)	31 (15.7)	82 (10.6) *
Others	59 (6.1)	10 (5.1)	49 (6.3)

*p < 0.05; ** p < 0.005; *** p < 0.0001

*Abbreviation*: *TCM* traditional Chinese medicine

### Identified drug-related problems

A total of 242 DRPs were identified, an average of 0.25 per patient (Table 3). Treatment Safety P2 was the major type of DRPs (106; 43.8%) followed by treatment effectiveness P1(78;32.2%). Within the treatment effectiveness P1 category, the effect of drug treatment not optimal P1.2 was the dominant category. Unnecessary drug-treatment P3.2 was the major category of Others P3.

**Table 3:** Types of drug-related problems and pharmacists’ interventions and outcomes according to the Pharmaceutical Care Network Europe DRP classification tool V9.00

Primary domain	Number	Frequency (%)
Types of drug-related problems		
Treatment effectiveness P1		
No effect of drug treatment P1.1	3	1.24
Effect of drug treatment not optimal P1.2	58	24.0
Untreated symptoms or indication P1.3	17	7.02
Treatment Safety P2		
Adverse drug event (possibly) occurring P2.1	106	43.8
Others P3		
Problem with the cost-effectiveness of the treatment P3.1	2	0.83
Unnecessary drug-treatment P3.2	56	23.1
Pharmacists’ interventions		
At prescriber level I1		
Prescriber informed only I1.1	1	0.2
Prescriber asked for information I1.2	13	2.5
Intervention proposed to prescriber I1.3	199	37.9
Intervention discussed with prescriber I1.4	28	5.3
At patient level I2		
Spoken to family member/caregiver I2.4	19	3.6
At drug level I3		
Drug changed to … I3.1	43	8.2
Dosage changed to … I3.2	42	8
Formulation changed to … I3.3	6	1.1
Instructions for use changed to … I3.4	69	13.1
Drug paused or stopped I3.5	59	11.2
Drug started I3.6	42	8
Other intervention or activity I4		
Side effect reported to authorities I4.2	4	0.8
Outcomes of interventions		
Not known O0		
Problem status unknown O0.1	0	0
Solved O1		
Problem totally solved O1.1	225	93.0
Partially solved O2		
Problem partially solved O2.1	0	0
Not solved O3		
Problem not solved, lack of cooperation of patient O3.1	2	0.8
Problem not solved, lack of cooperation of prescriber O3.2	15	6.2
Problem not solved, intervention not effective O3.3	0	0
No need or possibility to solve problem O3.4	0	0

*Abbreviations*: *P* problem, *I* intervention, *O* outcome

### Causes of drug-related problems identified

A total of 281 DRPs causes were identified (Table 4). Drug selection C1 was the primary cause of DRPs (124;44.1%) followed by dose selection C3(92;32.7%). Within the dose selection C3, dosage regimen too frequent C3.4 was the dominant subcategory followed by drug dose too high C3.2. Inappropriate drug according to guidelines/formulary C1.1was the major subcategory in the drug selection domain C1.

**Table 4:** Identified causes according to the Pharmaceutical Care Network Europe DRP classification tool V9.00

Primary domain	Cause of the problem	Total number = 281 (100.0%)
Drug selection C1	Inappropriate drug according to guidelines/formulary C1.1	33 (11.7)
	Inappropriate drug (within guidelines but otherwise contraindicated) C1.2	16 (5.7)
	No indication for drug C1.3	7 (2.5)
	Inappropriate combination of drugs, or drugs and herbal medications, or drugs and dietary supplements C1.4	15 (5.3)
	Inappropriate duplication of therapeutic group or active ingredient C1.5	14 (5.0)
	No or incomplete drug treatment in spite of existing indication C1.6	25 (8.9)
	Too many drugs prescribed for indication C1.7	14 (5.0)
Drug form C2	Inappropriate drug form (for this patient) C2.1	13 (4.6)
Dose selection C3	Drug dose too low C3.1	8 (2.8)
	Drug dose too high C3.2	33 (11.7)
	Dosage regimen not frequent enough C3.3	7 (2.5)
	Dosage regimen too frequent C3.4	37 (13.2)
	Dose timing instructions wrong, unclear or missing C3.5	25 (8.9)
Treatment duration C4	Duration of treatment too short C4.1	1 (0.4)
	Duration of treatment too long C4.2	1 (0.4)
Drug use process C6	Inappropriate timing of administration and/or dosing intervals C6.1	1 (0.4)
Patient related C7	Patient uses/takes more drug than prescribed C7.2	1 (0.4)
	Inappropriate timing or dosing intervals C7.7	13 (4.6)
	Patient administers/uses the drug in a wrong way C7.8	1 (0.4)
	Patient unable to use drug/form as directed C7.9	1 (0.4)
Patient transfer related C8	No medication reconciliation at patient transfer C8.1	2 (0.7)
	No updated medication list available C8.2	1 (0.4)
	Discharge/transfer information about medication incomplete or missing C8.3	2 (0.7)
	Patient has not received necessary medication at discharge from hospital or clinic C8.5	1 (0.4)
Other C9	Other cause; specify C9.2	9 (3.2)

*Abbreviations*: *C* cause

### Pharmacists’ interventions to solve the drug-related problems

A total of 525 interventions were suggested; an average of 2.2 interventions per DRP identified (Table 3). Most interventions occurred at the prescriber level I1 (241;45.9%) followed by at the drug level I3(238;45.3%). At the prescriber level, intervention proposed to prescriber I1.3 was the major subcategory; and instructions for use changed to … I3.4 was the major subcategory at the drug level, followed by at the drug paused or stopped I3.5. Total 480(91.4%) interventions were accepted and fully implemented by prescribers or patients, while 45(8.6%) interventions were not accepted by prescribing physicians. 225(93.0%) DRPs were totally solved, and 17(7.0%) DRPs were unresolved (Table 3). Among the not solved O3 domain, lack of cooperation of prescriber O3.2were the major causes of failed intervention outcome.

### Severity ratings of DRPs

The severity ratings of DRPs from low to high were category B (107;44.2%), category C (79;32.6%), category D (24;9.9%), category E (25;10.3%) and category F (7;2.9%). A total of 86.8% of the DRPs were rated at severity categories B to D (causing no patient harm). None of DRPs was implicated to cause death (subcategory I). Medications that caused harm mainly included antihypertensive agents, insulin, diuretics, anti-infective drugs, and nonsteroidal anti-inflammatory drugs. All-cause mortality in DRP and non-DRP groups were 0.

### Analysis of factors associated with the occurrence of DRPs

Patients with DRPs had a higher median age in comparison with those without DRPs (*p* < 0.005). The median length of hospital stay was 14.0(11.0,15.0) days, and patients with DRPs had a longer duration of hospital stay, but no statistically significant difference (*p* = 0.063) (Table 1).

More DRPs were identified in patients with cerebrovascular disease, hypertension, diabetes, infectious diseases, and atrial fibrillation. Patients with DRPs also exhibited a lower median creatinine clearance than patients without DRPs *p* < 0.05) (Table 1). The top 5 medication classes were antihyperlipidemic agents (86.4%), Traditional Chinese Medicine (85.2%), antiplatelet agents (81.9%), antihypertensive agents (71.0%) and digestive system medications (46.2%). Patients with DRPs exhibited greater use of antihypertensive agents, antidiabetic agents, anti-infective medications, anticoagulants, electrolytes, respiratory medications, and liver protective medications than patients without DRPs (Table 2). More patients with DRPs were placed on nasogastric feeding than patients without DRPs (17(8.6%) vs 13(1.7%), *p* < 0.0001). Of these nasogastric feeding in patients with DRPs, 13 DRPs involved inappropriate drug forms mainly included enteric coated, sustained, and controlled release dosage forms such as aspirin enteric-coated tablet, nifedipine controlled-release tablet. Patients with DRPs took a greater number of medications than patients without DRPs (13.5 (11.0,17.0) vs 11.0(9.0,14.0), *p* < 0.0001).

Before proceeding to multiple logistic regression analysis, 16 variables (*p* < 0.1) in the univariate analysis (including age, smoke, creatinine clearance, number of medications used, nasogastric feeding, diabetes, cerebrovascular diseases, hypertension and so on) were assessed for multicollinearity. The results showed that all VIF values were less than 10, indicating the absence of multicollinearity. In multiple logistic regression analysis, factors of creatinine clearance, number of medications used, nasogastric feeding, diabetes, infectious diseases associated with more frequent DRPs (*p* < 0.05) (Table 5).

**Table 5:** Multiple logistic regression analysis of factors associated with the occurrence of DRPs

Factors	Adjusted OR (95% CI)	*p* value
Age	1.000 (0.982–1.019)	0.997
Sex	1.423 (0.892–2.270)	0.138
Smoke, currently	1.098 (0.706–1.709)	0.678
Creatinine clearance	0.993 (0.986–0.999)	0.030
Number of medications used	1.082 (1.025–1.141)	0.004
Nasogastric feeding	3.882 (1.033–14.587)	0.045
Diabetes	1.835 (1.227–2.744)	0.003
Cerebrovascular diseases	0.829 (0.520–1.320)	0.429
Hypertension	1.329 (0.834–2.120)	0.232
Infectious diseases	3.772 (1.822–7.808)	< 0.001
Atrial fibrillation	1.566 (0.452–5.424)	0.479
Anticoagulants	1.181 (0.454–3.070)	0.734
Respiratory medications	0.502 (0.220–1.144)	0.101
Length of hospital stay	0.972 (0.924–1.024)	0.286
Liver dysfunction	1.321 (0.763–2.286)	0.320
Electrolytes	1.399 (0.849–2.305)	0.187

## Discussion

This is the first retrospective study conducted in China to categorically evaluate DRPs using PCNE classification, and to identify factors associated with the occurrence of DRPs in the neurology unit of a tertiary care and academic teaching hospital in China. DRPs were relatively common in the neurology unit, an average of 0.25 per patient. This number was close to that in two studies, 0.39 from one study conducted in the neurology ward in Switzerland by pharmacists [18], and 0.29 from the other study done in the inpatient ward and intensive care unit of the department of neurology in Egypt [16]. However, the average number of DRPs per patient in this study was lower than the following two studies: (1) the Brazilian study (1.26 DRPs per patient) using the trigger DRP classification [15], (2) the Iran research (1.37 DRPs per patient) using the modified PCNE DRP classification V5.01 [17]. These studies showed a significantly different mean number of DRPs per patient in different hospitals. Study population size and setting, characteristics of the patient population, study duration, definition of DRPs, types of classification systems, patterns of medication use, and differences of clinical guidelines may contribute to the variations of DRPs prevalence among studies [20–22].

Multiple logistic regression showed that creatinine clearance, number of medications used, nasogastric feeding, diabetes, and infectious diseases were factors associated with DRPs. The findings that age and length of hospital stay were not factors predicting the occurrence of DRPs in our analysis were different from previous studies [15, 16, 23]. It was worth noting that whether patients were fed through nasogastric feeding was an important factor associated with DRPs. Since a significant proportion of patients admitted to the neurology unit may require nasogastric feeding, the use of modified-release medication products can be problematic [24]. Modified-release drug products such as nifedipine controlled-release tablets, felodipine sustained-release tablets, and aspirin enteric-coated tablets when crushed may alter the release profile and location, which may affect its effectiveness and safety. However, because the doctors lacked knowledge on differences of various types of dosage forms, patients that were fed through nasogastric feeding were more likely to have DRPs [21].

In our study, treatment safety was the major type of DRPs. The result was different compared to the Brazilian and Egypt study, where the untreated condition was the major type of DRP identified. The result indicates the unique role that clinical pharmacists in China play in ensuring the safe use of medications for patients in the neurology unit. Drug selection and dose selection accounted for approximately 80% of the causes of the DRPs in our study. Inappropriate drugs according to guidelines/formulary was the major subcategory in the drug selection domain. For example, ischemic stroke patients took clopidogrel bisulfate tablets in combination with omeprazole sodium enteric-coated tablets leading to a decrease in the efficacy of clopidogrel. Hypertensive patients with hyperuricemia were treated with hydrochlorothiazide. This demonstrates that clinical pharmacists play an influential role in optimizing medication therapy in the MDT. Within the dose selection, dosage regimen too frequent was the dominant subcategoryfollowed by drug dose too high. Many medications require dose adjustments for patients with old age and kidney or liver function impairment. This indicates the importance of clinical pharmacists to conduct prospective prescription reviews to ensure correct dosage and frequency of medication.

In our study, 525 interventions were suggested by the clinical pharmacist with a mean of 2.2 interventions per DRP, and interventions were highly accepted (91.4%). Previous studies showed a significantly different acceptance rate varying from 41.91 to 90.9% [15–18, 25]. The highest rate of acceptance so far demonstrates that pharmacist interventions were highly useful for physicians and a sufficient relationship of trust between physicians and pharmacists. The fact that 86.8% of DRPs were rated at severity categories B to D (causing no patient harm) further proves the importance of clinical pharmacists in preventing medication errors and ensuring medication safety.

Our study has the following limitations: (1) this was a single-center study and patient populations admitted into the neurology unit could not include all neurologic diseases due to specializations of the department, so our findings may not be generalizable to other hospitals and neurology units in China, and (2) medication review was performed by one clinical pharmacist, and (3) our study did not assess the relationship between patients’ long-term outcomes and the resolution of DRPs.

## Conclusion

This study indicates that the prevalence of DRPs is relatively common in Chinese neurology patients. Treatment safety is the major type of DRPs. Improving clinical pharmacy services in neurology unit could contribute to rational medication use and ensure patient safety.

## Data Availability

The data used to support the findings of this study are included within the article. If further information is required, the authors will furnish the additional supporting data.
